# Alkylation and Carbamylation Effects of Lomustine and Its Major Metabolites and MGMT Expression in Canine Cells

**DOI:** 10.3390/vetsci2020052

**Published:** 2015-04-24

**Authors:** Thushara Chakkath, Sidonie Lavergne, Timothy M. Fan, David Bunick, Levent Dirikolu

**Affiliations:** 1 Department of Comparative Biosciences, College of Veterinary Medicine, University of Illinois, Urbana, IL 61802, USA; E-Mails: chakkat1@illinois.edu (T.C.); slavergn@illinois.edu (S.L.); dbunick@illinois.edu (D.B.); 2 Department of Veterinary Clinical Medicine, College of Veterinary Medicine, University of Illinois, Urbana, IL 61802, USA; E-Mail: T-fan@illinois.edu

**Keywords:** lomustine, trans-4-hydroxylomustine, cis-4-hydroxylomustine, lymphoma, MGMT

## Abstract

DNA Alkylation is thought to be the reason for the efficacy of lomustine while carbamylation has been implicated as the cause for the side effects seen with lomustine treatment such as hepatotoxicity. In the alkylation study we show that lomustine and its metabolites form similar levels of the DNA adducts N^7^ hydroxyethylguanine and O^6^ hydroxyethyldeoxyguanosine. In terms of carbamylation, lomustine showed greater extent of carbamylation in the canine hepatocytes and lymphoma cell lines. The DNA repair enzyme O^6^ methylguanine DNA methyltransferase (MGMT) causes resistance of tumor cells to bifunctional nitrosourea, like lomustine. There is no data available regarding MGMT expression/activity in canine cells or tissues. Our study shows that there is low MGMT activity in the canine lymphoid cell line 17–71 while the GL-1 cells did not show any detectable enzyme activity or mRNA expression. The MGMT enzyme activity measured in canine hepatocytes is about 250–350 fmol/mg protein as compared to about 90 fmol/mg protein in 17–71 cells. We also show that MGMT mRNA expression in 17–71 cells and canine hepatocytes positively correlates with its enzyme activity in these cells.

## 1. Introduction

It is known that alkylating agents such as nitrosoureas decompose at physiological conditions to yield alkylating and carbamylating intermediates [1], however the relative contribution of these events to the toxicity and therapeutic effects are unclear [2]. These compounds are electrophilic in nature and attack the nucleophilic sites on the DNA to form alkylated products [3]. The N^7^ position of guanine is the most reactive site [3,4] with all alkylating agents and hence about 70%–90% of the adducts are formed at this position [5,6]. However the relative mutagenicity and carcinogenicity of alkylating agents correlate with the reagents ability to bind to the oxygen sites [7,8]. Chloroethylating compounds like lomustine form adducts at the O^6^ position of guanine [5]. If not repaired, these adducts form DNA interstrand cross links [3] which may lead to double strand breaks and ultimately, apoptosis of the cell [9].

Decomposition of lomustine in aqueous medium produces chloroethyl carbonium ions which is an alkylating intermediate and also cyclohexyl isocyanate which is a carbamylating agent [10,11,12]. It is believed that the dominant role of carbamylating activity is in determining the toxicity of compounds which may be manifested as side effects [2]. The isocyanate intermediates formed by the breakdown of lomustine react with the lysine residues in protein leading to post translational modification of proteins [13]. This could result in partial or complete loss of protein function [14].

The DNA protective enzyme MGMT is known to play a critical role in the defense against alkylating compounds which produce the O^6^ alkylguanine lesion. Repair of the O^6^ alkylguanine adduct via MGMT is a rapid single step process in which the offending alkyl group is transferred to the cysteine residue of MGMT thereby restoring the affected DNA [5,15,16]. The MGMT level is highly variable among individuals and also among the various tissues in the same individual [17]. A long term study of MGMT in peripheral blood mononuclear cells (PBMC) from healthy human subjects showed that there is a 7 fold variation in MGMT activity between subjects with very little change over time [18].

Due to its functional role in DNA repair, MGMT may have a significant impact on the outcome of cancer therapy with alkylating agents. Certain tumors that are high expressers of MGMT were found to be less sensitive to treatment with nitrosoureas while tumors that were deficient in MGMT or showed relatively low expression responded better to nitrosourea therapy [17]. MGMT levels in normal tissues may also have significant impact on the choice of therapy to be used. A dose limiting side effect of most nitrosourea compounds is myelotoxicity, and it is thought that this could be due to the low MGMT expression in bone marrow cells [16,19]. There is data indicating that MGMT expression is inducible (via transcriptional activation) by alkylating agents, X-ray or other DNA damaging agents [20]. Studies in rat liver cells have shown a 2–5 fold MGMT gene induction with maximum induction happening around 24 to 48 h after exposure to X-ray or the alkylating agent, methylnitronitrosoguanidine (MNNG) [20,21].

In our previously published cytotoxicity study [22], lomustine and its metabolites showed similar degree of cell killing in the canine lymphoid cell lines 17–71 and GL-1, and canine hepatocytes. As alkylation is thought to be the main reason for the cytotoxic effects of these drugs [3], we wanted to investigate if the compounds have similar pattern of alkylation. It is believed that the concentration of an internal dosimeter such as DNA adducts is a more accurate measure of the amount of mutagen reaching the target site than the applied dose [7]. In a recent study published, it was shown that N^7^ hydroxyethyl guanine and O^6^ hydroxyethyldeoxyguanosine are some of the main DNA alkylation products formed after exposure to chloroethylating agents [23]. It is reported that the cis-4 and trans-4 metabolites of lomustine may have reduced toxic effects relative to lomustine [24] and therefore the metabolites might have advantage over lomustine for clinical use [25]. Carbamylation of proteins is believed to contribute to the side effects seen with lomustine [2].

The aim of this study was to compare the alkylation and carbamylation potential of lomustine and its trans-4 and cis-4 metabolites in canine lymphoma cell lines and hepatocytes. The secondary aim was to evaluate the MGMT expression and enzyme activity in canine lymphoid cell lines and primary canine hepatocytes as we tried to correlate the MGMT levels with the cytotoxicity of lomustine and its metabolites in these cells and also the level of DNA adducts formed by these compounds.

## 2. Materials and Methods

### 2.1. DNA Adduct Detection Assay

The O^6^ hydroxyethyldeoxyguanosine and N^7^ hydroxyethylguanine adducts were measured in canine lymphoid cell lines 17–71 and GL-1 [26,27] and primary canine hepatocytes (isolated from six dogs that were euthanized at the Champaign county animal control facility) after exposure to lomustine, trans-4 and cis-4 by the LC/MS/MS method as mentioned below. The cell lines were grown and maintained at 37 °C in 5% CO_2_, and cultures were passaged as necessary to maintain high cell viability (above 90%) in culture media Dulbecco’s modified Eagle medium (DMEM) supplemented with 10% fetal bovine serum (FBS) and 1% penicillin/streptomycin.

#### 2.1.1. Isolation of Canine Hepatocytes

Isolation of canine hepatocytes was performed as previously described [28] with few modifications. Liver samples (25 gms) from freshly euthanized dogs were placed in PBS containing 1% penicillin/streptomycin, finely minced, washed a couple of times with PBS, and transferred into 100 mL of cold collagenase solution (Collagenase II, 300 units/mg, 500 ng/mL in hank’s balanced salt solution (HBSS)-Hepes buffer) and stirred for 10 min. At the end of incubation, 10 mL of FBS and 50 mL of ice cold HBSS were added. The suspension was filtered through a cell strainer (100 micron pore size) and the filtrate was centrifuged at 50 *g* for 5 min at 4 °C. The pellet was washed twice with PBS with 1% penicillin/streptomycin and centrifuged twice at 150 *g* for 2 min each and resuspended in 5 mL PBS. The cell suspension was layered over 15 mL cushion of 60% Percoll in PBS and centrifuged at 70 *g* at 4 °C for 5 min. The pellet was washed again with PBS and cell viability was determined by Trypan blue dye exclusion test. The cells were then suspended in DMEM (supplemented with 10% FBS and 1% penicillin/streptomycin) at a density of 0.6 million/mL and incubated at 37 °C for 48 h to acclimatize and form a monolayer. After 48 h the media was replaced by hepatocyte maintenance media (Triangle Research, NC, USA). The maintenance media was changed every day.

#### 2.1.2. Sample Preparation

For this experiment, 10^6^ cells were used per sample. The cells were exposed to fresh media (hepatocyte maintenance media for the canine hepatocytes and DMEM supplemented with 10% FBS and 1% penicillin/streptomycin for the canine cell lines 17–71 and GL-1) containing varying concentrations of lomustine, trans-4 or cis-4 metabolites (300, 1000, 3000, 10,000 and 50,000 ng/mL) for 4 h. At the end of 4 h, the DNA was isolated using Qiagen blood mini kit (Qiagen). The extracted DNA was lyophilized and concentrated to 100 µg and then digested as follows: per 50 μL DNA, 10 μL of 0.5 M Tris (pH 7.3), 2 μL of 1 M MgCl_2_, 10 μL DNAse I (5 mg/mL, 2303 units/mg, Worthington), 10 μL of snake venom phosphatase (5 mg/mL, 36 units/mg, Worthington) and 4 μL of bacterial alkaline phosphatase (5 mg/mL, 35 units/mg, Worthington) was added final volume adjusted to 150 μL with deionized water. The mixture was incubated overnight at 37 °C and then heated to 100 °C for 90 s to inactivate the enzymes. The samples were briefly centrifuged to sediment the proteins and the supernatant was analyzed by LC/MS/MS. For the cell lines, the experiment was done two separate times, with two technical replicates each. For the canine hepatocytes, the experiment was done once with duplicate samples.

The 4 h time point for measuring adducts after drug exposure was based on a published study that looked at DNA alkylation products after exposure to lomustine and adducts were measured between 1 and 6 h. The published results showed that the adducts were formed rapidly and were detected at 1 h after drug exposure [23]. In our initial experimentation, the 1 h samples showed very low adduct level at the 10,000 ng and 50,000 ng concentrations and no adducts were detected at the lower drug concentrations. Even in the 4 h samples, the adducts were only detected at the 10,000 ng and 50,000 ng but the response was greater compared to the 1 h samples. Hence we chose to detect the adducts at 4 h. The N^7^ adduct was detected at the concentration of 10,000 ng/mL and above while the mutagenic adduct O^6^ hydroxyethyldeoxyguanosine was only detected at 50,000 ng/mL drug concentration. Therefore the 10,000 ng/mL and 50,000 ng/mL concentrations were used to compare the adducts formed by the three drugs.

### 2.2. LC/MS/MS Method for Detection of DNA Adducts

An LC/MS/MS method was developed to quantify the O^6^ hydroxyethyldeoxyguanosine and N^7^ hydroxyethylguanine adducts in the DNA samples.

#### 2.2.1. Standards Preparation

Pure standards of O^6^ hydroxyethyldeoxyguanosine and N^7^ hydroxyethylguanine were purchased from Toronto research company (Toronto, ON, Canada). Deuterated O^6^ methylguanine (D3-O^6^ methylguanine) was used as internal standard (Sigma Aldrich, St. Louis, MO, USA). Standards were dissolved in methanol to prepare stock solutions. Standard curve range was 0.1–10 ng for each compound and 50 ng of internal standard was added to each sample. The final volume was adjusted to 150 µL with water.

#### 2.2.2. LC/MS/MS Conditions

Samples were analyzed with the QTRAP 5500 LC/MS/MS system (AB Sciex, Foster City, CA, USA) with the 1200 series HPLC system (Agilent Technologies, Santa Clara, CA, USA) including a degasser, an autosampler, and a binary pump. The LC separation was performed on an Agilent SB-Aq column (4.6 mm × 50 mm, 5 µ) (Santa Clara, CA, USA) with mobile phase A (0.1% formic acid in water) and mobile phase B (0.1% formic acid in acetonitrile). The flow-rate was 0.35 mL/min. The linear gradient was as follows: 0–1 min, 100% A; 8–11 min, 25% A; 12–18 min, 100% A.

**Figure 1:**
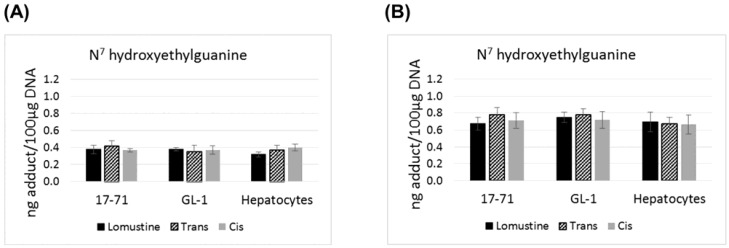
Graphs showing the N^7^ hydroxyethylguanine adduct formed after treatment with lomustine, trans-4-hydroxylomustine and cis-4-hydroxylomustine metabolites at 10,000 ng/mL (**A**) and 50,000 ng/mL (**B**) concentration in 17–71 and GL-1 cells and hepatocytes from 6 dogs (*n* = 6).

The autosampler was set at 5 °C and the injection volume is 5 µL. Positive mass spectrometry was achieved with electrospray ionization (ESI). The ion spray voltage was 5500 V. The source temperature was 400 °C. The curtain gas, ion source gas 1, and ion source gas 2 were 32, 65, and 50, respectively. Multiple reaction monitoring (MRM) was used to quantify O^6^ hydroxyethyldeoxyguanosine (m/z 312.2 àm/z 110.1), N^7^ hydroxyethylguanine (m/z 196.1 àm/z 110.1), and the internal standard deuterated O^6^ methylguanine (m/z 169.1 àm/z 70.1) (Figure 1).

### 2.3. Carbamylation Assay

Carbamylation was measured in primary hepatocytes harvested from six dogs euthanized at the Champaign county animal control facility (hepatocyte isolation procedure as mentioned above) and canine cell lines 17–71 and GL-1 after treatment with lomustine and its metabolites using Oxyselect™ carbamylation ELISA kit.

Cells were counted and 10^6^ cells were used per sample. The cells were exposed to fresh media (hepatocyte maintenance media for the canine hepatocytes and DMEM supplemented with 10% FBS and 1% penicillin/spreptomycin for the canine cell lines 17-71 and GL-1) containing varying concentrations of lomustine, trans-4 or cis-4 metabolites (300, 1000, 3000 and 10,000 ng/mL). Carbamylation was measured at 4 h after exposure to drugs as per the ELISA kit instructions. Carbamylation is a rapid process occurring within a few minutes and previously published studies have measured carbamylation at similar time points (2, 19). Initial experimentation did not show any carbamylation at 300, 1000 and 3000 ng/mL drug concentration. Thus 10,000 ng/mL concentration was used to compare the carbamylation effects of the three drugs. For the cell lines, the experiment was done two separate times, with two technical replicates each. For the canine hepatocytes, the experiment was done once with duplicate samples.

### 2.4. MGMT mRNA Expression in 17–71 and GL-1 Cells and Canine Primary Hepatocytes

The canine cell lines 17–71 and GL-1 were grown and maintained at 37 °C in 5% CO_2_, and cultures were passaged as necessary to maintain high cell viability (above 90%) in culture media DMEM supplemented with 10% FBS and 1% penicillin/streptomycin. For the MGMT gene induction study, MGMT gene expression was measured in canine cells at 6, 12, 24 and 48 h after exposure to varying concentrations of lomustine (1000, 3000 and 12,000 ng/mL) to study MGMT induction. The cells were only treated with 12,000 ng/mL concentration of trans-4 and cis-4, based on the initial results obtained after lomustine treatment study. Untreated samples were used to measure basal MGMT mRNA expression in these cells. Hepatocytes were isolated from six dogs euthanized at the Champaign county animal control facility.

#### MGMT mRNA Expression Assay

The total RNA from samples was extracted using RNeasy mini kit (Qiagen). Relative expression levels of the MGMT gene was carried out using Taqman Real-Time quantitative real-time polymerase chain reaction (RT-PCR (QPCR)) analysis on an ABI Prism 7500 thermal cycler. An ABI canine MGMT specific Taqman probe (cf02626419_m1) spanning the exon 1/2 junction was used for all analyses. HPRT-1 was used as internal reference for normalization (ABI Taqman probe cf02626258_m1). For this analysis, 1 µg aliquot of total RNA from a given sample was converted to cDNA using Superscript III first strand synthesis system (Life technologies, Carlsbad, CA, USA). The resulting cDNA was used in separate Taqman QPCR analyses, each including triplicate technical repeats. Expression comparisons was made between treated and untreated (control) samples and also between different cell types. The mRNA expression was measured two separate times in all samples (canine cell lines and hepatocytes) with two technical replicates each time. In order to ascertain that the Taqman assay is able to detect the gene expression correctly, MGMT gene expression was measured in three canine glioma cell lines J3T-Bg, SDT-3 and G06A.

### 2.5. MGMT Enzyme Activity in 17–71 and GL-1 Cells and Canine Hepatocytes

A fluorometric oligonucleotide assay was used for measuring MGMT enzyme activity [29]. The following 18 base pair (bp) 5'hexachlorofluoroscein phosphoramitide (HEX) labelled oligonucleaotide (oligo) containing O^6^-methylguanine lesion within a *Pvu*II restriction site and its complementary oligo were custom made (Sigma Aldrich).

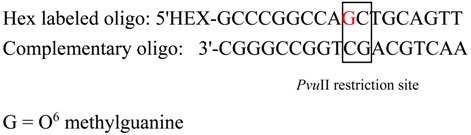



Oligonucleotide strands were annealed to complementary strands in 10 mM Tris–HCl, 1 mM EDTA, 33 mM NaCl, pH 8.0, by heating to 90 °C for 2 min to denature and cooling to room temperature overnight. The 17–71 and GL-1 cells and hepatocytes were maintained in culture media as mentioned above. Cells (10^6^) were harvested and resuspended in serum-containing culture medium (DMEM), then centrifuged at 1500 *g* for 5 min.

The cells were washed with 10 mL of PBS and recentrifuged, then resuspended in 0.5 mL assay buffer (50 mM Tris–HCl, 1 mM DTT, 0.5 mM EDTA, 5% glycerol, pH 8.0) and kept on ice. Protein from cells was extracted by pulse-sonication on ice five times for 5 seconds each, then centrifuged at 14,000 *g* at 4 °C for 30 min. The total protein was quantitated by measuring absorbance at 595 nm using the Pierce bicinchoninic acid (BCA) kit reagent. To measure the MGMT activity the 200 fmols of HEX labeled oligo was incubated with cell protein (500 µg) at 37 °C for 2 h. The oligo was extracted by phenol:chloroform:isoamyl alcohol (25:24:1) extraction and using sodium acetate and ethanol with tRNA (10 µg) as a carrier. This was vortex mixed for 5 s and then centrifuged at 12,000 g for 5 min at room temperature. The upper aqueous phase was transferred to a new tube and the purified oligo was digested with a restriction enzyme *Pvu*II (Promega) for 2 h in a total reaction mixture of 20 µL following manufacturer’s instructions. *Pvu*II cleaves the oligo at the O^6^-methylguanine lesion producing two short fragments (10 bp and 8 bp). After adding formamide loading buffer (10 µL), the samples were denatured by heating at 95 °C for 5 min. Sample solutions (15 µL) were applied to a pre-warmed 20% polyacrylamide gel containing 8 M urea in 1× Tris–borate EDTA (TBE) buffer and run at 50 mA for 15 min. Detection and quantitation of the fluorescent HEX labeled oligonucleotides was done by using a fluorescent imaging system (Typhoon 9410 Variable Mode Imager).

The MGMT enzyme activity was quantified by comparing the intensity of the bands (18 bp) between the *Pvu*II treated and *Pvu*II nontreated (control) samples. The MGMT activity was calculated by comparing the intensity (density volume obtained from the fluorescent reader) of the top band (18 bp) in the samples with that in the controls. The density of the control band was considered as 100%. The deficit in intensity in the sample bands compared to the control bands represents the amount of cleavage which corresponds to the MGMT enzyme activity. For the cell lines and hepatocytes, the experiment was done with duplicate samples.

For measuring the lower limit of detection (LOD) and limit of quantitation (LOQ) of the method, serial dilution of the HEX labelled oligo was made from 1–200 fmols in deionized water and the fluorescent intensity was measured and plotted against the oligo concentration. The lowest concentration showing 3 times the fluorescent intensity compared to the blank sample was chosen as the LOD and the lowest concentration with CV less than or equal to 15% was chosen as the LOQ of the method.

## 3. Statistical Analysis

Statistical analysis was done using SPSS (IBM, Armonk, NY, USA). Descriptive statistics were produced for all continuous variables. Mean and standard deviation were calculated. Normality of data was assessed using the Shapiro-Wilks test. For alkylating and carbamylation assays, paired *t*-tests were performed to evaluate differences between technical repeats of each drug and one way ANOVA and Tukey’s post hoc test were performed to evaluate the between drug differences. Kruskal-Wallis one way ANOVA was used to compare the means of the drug treated and non-treated groups at different time points in the MGMT gene induction study. Correlation between MGMT mRNA expression and enzyme activity was evaluated by Pearson’s correlation. The *p* value was set at 0.05.

## 4. Results

**Figure 2:**
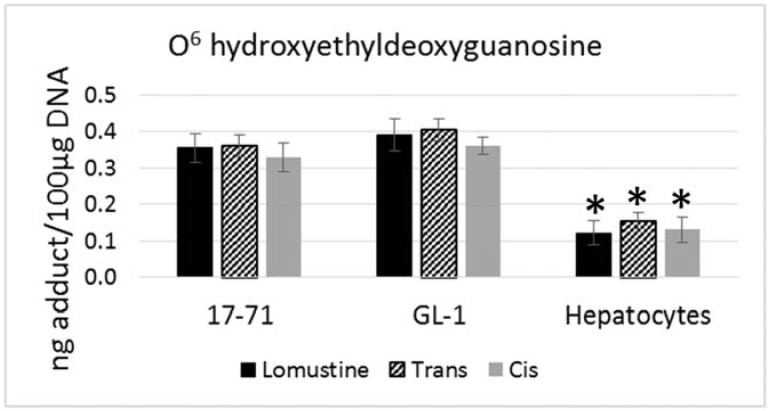
Graph showing the O^6^ hydroxyethyldeoxyguanosine adduct formed after treatment with lomustine, trans-4-hydroxylomustine and cis-4-hydroxylomustine metabolites at 50,000 ng/mL concentration in 17–71 and GL-1 cells and hepatocytes from six dogs (*n* = 6). * denotes statistical significance as compared to the 17–71 and GL-1 cells. *p* = 0.05.

There is no statistically significant difference in the levels of N^7^ hydroxyethylguanine and O^6^ hydroxyethyldeoxyguanosine adducts formed by lomustine, trans-4 or cis-4. The N^7^ hydroxyethylguanine adduct was detected at similar level in 17–71, GL-1 and the hepatocytes from six dogs after exposure to lomustine, trans-4 and cis-4 at 10,000 ng/mL and 50,000 ng/mL concentration (Figure 1). However the mutagenic adduct O^6^ hydroxyethyldeoxyguanosine was detected at a significantly lower level in the hepatocytes (2–3 fold) as compared to the cell lines when treated with the three drugs at 50,000 ng/mL concentration (Figure 2).

**Figure 3:**
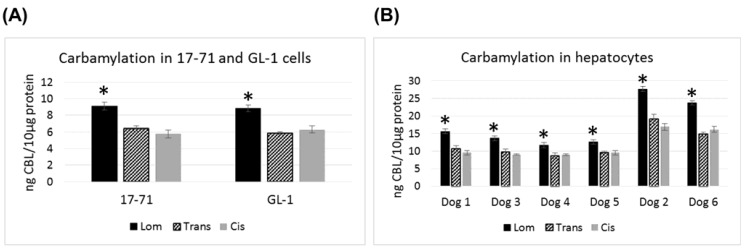
Graphs showing the carbamylation induced by lomustine, trans-4-hydroxylomustine and cis-4-hydroxylomustine at 10,000 ng/mL concentration in 17–71 and GL-1 cells (**A**) and hepatocytes from six dogs (**B**). * indicates statistical significance compared to trans-4-hydroxylomustine and cis-4-hydroxylomustine. *p* = 0.05.

In the canine cell lines 17–71 and GL-1 and in the canine hepatocyte samples, lomustine caused significantly greater carbamylation as compared to its metabolites (Figure 3). Both the metabolites showed similar carbamylation levels. In all the six canine hepatocyte samples tested, the carbamylation caused by all the three drugs was 1.5–2 fold greater than that seen in 17–71 and GL-1 cells. Among the six dog hepatocyte samples, two dogs showed about 2 fold higher level of carbamylation compared to the others with lomustine, trans-4 and cis-4.

**Figure 4:**
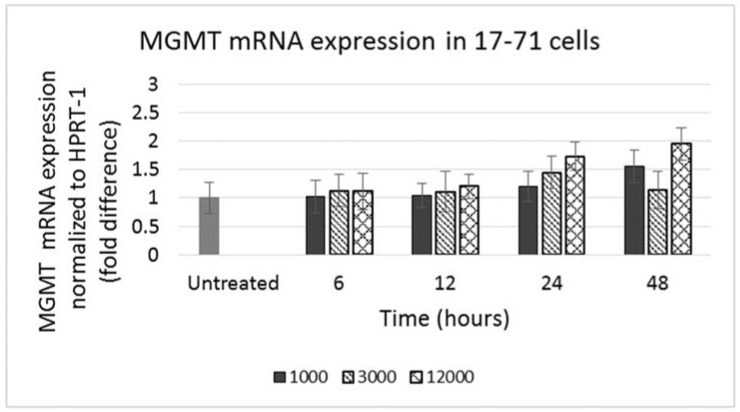
Graph showing MGMT mRNA expression in 17–71 cells before (untreated) and after treatment with lomustine at 1000, 3000 and 12,000 ng/mL and incubated for 6, 12, 24 and 48 h.

**Figure 5:**
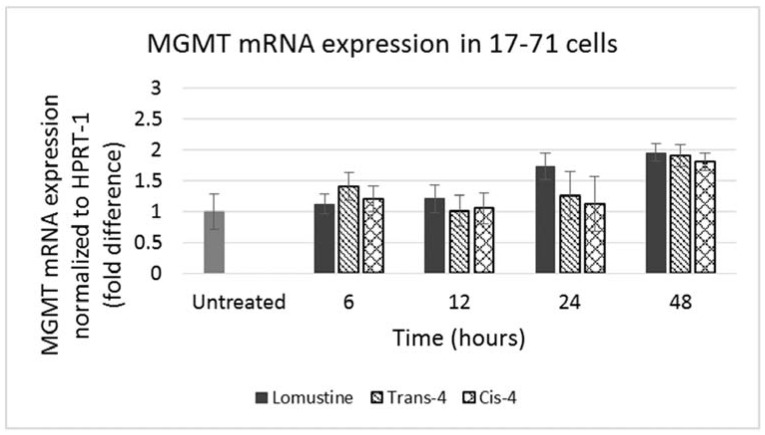
Graph showing MGMT mRNA expression in 17–71 cells before (untreated) and after treatment with trans-4-hydroxylomustine and cis-4 hydroxylomustine at 12,000 ng/mL concentration and incubated for 6, 12, 24 and 48 h.

GL-1 cells did not show any MGMT mRNA expression in untreated samples. When GL-1 samples were treated with lomustine, trans-4 and cis-4 at 1000, 3000 and 12,000 ng/mL and incubated for 12, 24 and 48 h, still no MGMT mRNA expression was detected. In the 17–71 cells, a low basal level of MGMT expression was seen in the untreated samples (Figure 4). Upon treatment with various concentrations of lomustine, trans-4 and cis-4 and incubated for different time points, no statistically significant difference was seen in any of the drug treated samples at any time point as compared to the vehicle control (Figure 4 and Figure 5).

**Figure 6:**
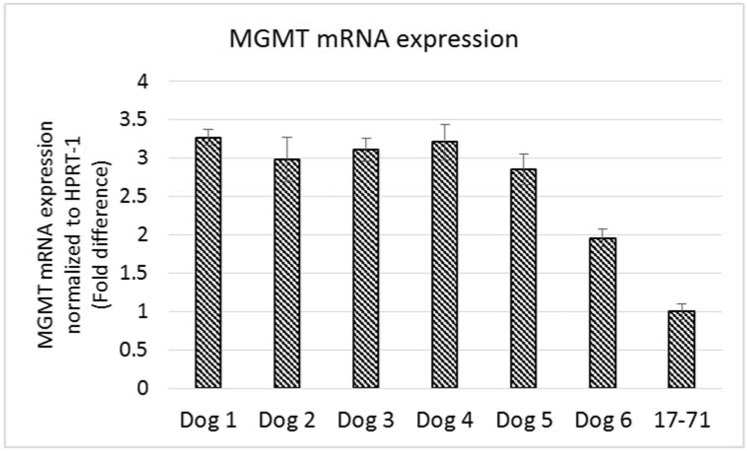
Graphs showing the MGMT mRNA expression in canine lymphoma cell line 17–71 cells and primary hepatocytes from six dogs.

The MGMT gene expression measured in the hepatocytes of the six dogs was 2-3 fold greater than that seen in the 17–71 cells (Figure 6). The variation in MGMT mRNA expression among the six dogs was low except for one dog which showed a lower MGMT level. Two of the glioma cell lines SDT-3 and J3G-Bg showed high MGMT mRNA expression (see supplemental data). The results show that the Taqman qPCR assay is able to detect higher transcript levels of MGMT gene and that the previously measured low MGMT gene expressions in 17–71 cells are indeed true values.

**Figure 7:**
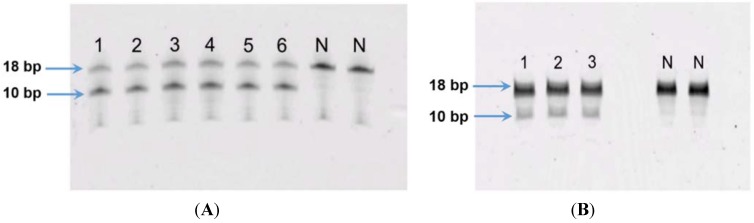
(**A**) MGMT activity in canine lymphoma cell line 17–71: lanes 1–3 represent replicate oligo samples treated with 500 μg cell protein from 17–71 cells. Lanes labeled “N” are untreated oligo samples (control). Upper and lower bands represent 18 bp substrate and 10 bp cleavage product, respectively; (**B**) MGMT activity in primary hepatocytes obtained from six dogs: lanes 1–6 represent oligo samples treated with 500 μg cell protein from hepatocytes obtained from six dogs. Lanes labeled “N” are untreated oligo samples (control). Upper and lower bands represent 18 bp substrate and 10 bp cleavage product, respectively.

The LOD of the fluorometric MGMT enzyme activity assay was 5 fmols while the LOQ was 10 fmols (see supplemental data). For GL-1 and 17–71 cell line, using 100 µg and 200 µg of cell protein did not show any MGMT activity. In order to ascertain the functionality of the assay, human recombinant MGMT (Cayman chemicals) was used as a positive control. The results showed that with increasing concentration of MGMT, greater cleavage of the oligo was seen (see supplemental data). Thus the protein amount in the assay was increased to 500 µg and in the 17–71 samples showed visible cleavage of the oligo (Figure 7A). The GL-1 cell lines did not show any MGMT enzyme activity even when 500 µg cell protein was used for the assay.

**Figure 8:**
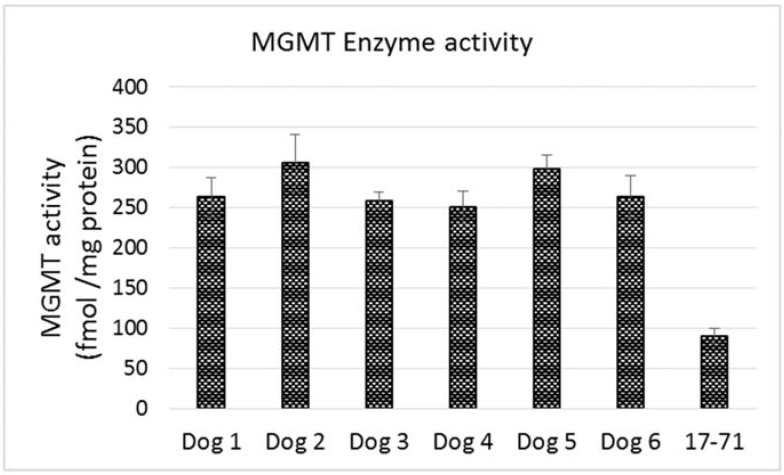
Graph showing MGMT enzyme activity in the primary hepatocytes obtained from six dogs and in the canine lymphoma cell line 17–71.

**Figure 9:**
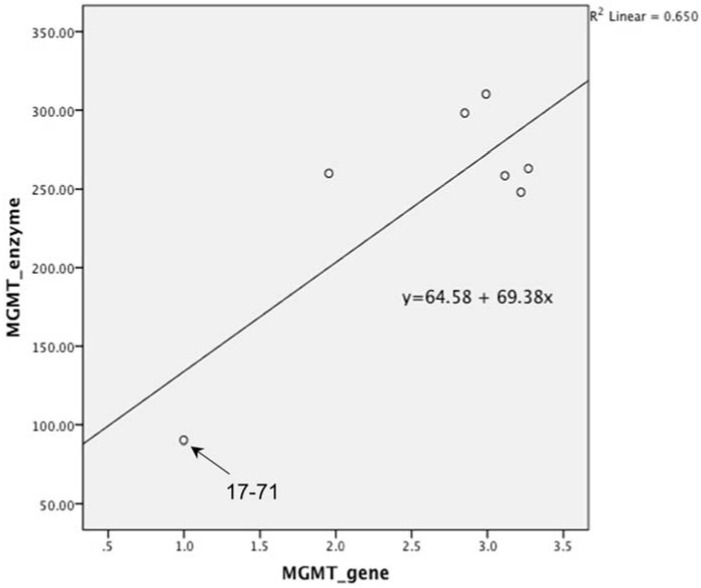
Graph showing correlation between MGMT gene expression and enzyme activity in canine lymphoma cell line 17–71 and the hepatocytes obtained from six dogs. Pearson’s correlation was 0.806 which was highly significant with *p* value of 0.028. (*p* value set at 0.05).

The MGMT activity in the 17–71 cells was about 90 fmols/mg protein (range 83–96 fmols/mg protein, SD 10.11). In the GL-1 cell line, as no MGMT gene expression was detected, consequently, MGMT enzyme activity was also absent. The enzyme activity measured in the hepatocytes was greater than in 17–71 cells, proportional to the mRNA levels. The MGMT enzyme activity measured in canine hepatocytes is about 250–350 fmols/mg protein (Figure 7B and Figure 8). There was good correlation between MGMT gene expression and enzyme activity in the 17–71 and the canine hepatocytes (Figure 9).

## 5. Discussion

In our study we found no significant difference in the N^7^ hydroxyethylguanine and O^6^ hydroxyethyldeoxyguanosine adduct level formed by lomustine, trans-4 and cis-4 which correlates with the similar cytotoxicity of these compounds. The mutagenic O^6^ adduct was higher in the canine cell lines 17–71 and GL-1 as compared to the hepatocytes which may indicate either lower adduct formation or rapid repair in canine hepatocytes. Rapid repair may be a possibility since all the canine hepatocyte samples showed greater MGMT level. It can be speculated that as MGMT is a DNA repair enzyme, an enhanced repair of O^6^ adducts could contribute to the reduced sensitivity of hepatocytes to the alkylating action of lomustine and its metabolites.

It should however be noted that we have only detected the levels of two individual adducts after drug exposure. The cell killing action of nitrosoureas may be orchestrated by the formation and cumulative action of multiple adducts that these drugs are capable of forming. Also, in case of chloroethylating agents like lomustine, cross linking of DNA is known to be harmful lesion that can lead to double stand breaks and ultimately apoptosis [13]. Thus in our alkylation study, measuring the two adducts can act only as a molecular dosimeter of therapeutic response and not a measure of the full extent of alkylation caused by the drugs.

Carbamylation was measured in the canine lymphoma cell lines and hepatocytes by an ELISA kit. The assay measures the amount of CBL in the samples. Our results show that lomustine causes greater carbamylation as compared to its metabolites in canine cell lines and hepatocytes. All the three compounds caused greater carbamylation in hepatocytes as compared to the canine cell lines. Also hepatocyte samples from two dogs showed greater carbamylation following treatment with lomustine and its metabolites compared to the others. But it should be noted that all of the dog hepatocyte samples showed no cytotoxicity to lomustine or its metabolites up to six days of incubation. Hence it is unlikely that greater carbamylation in the hepatocytes in the two dogs would lead to cell death in our experiments, although it may be inhibiting various proteins in these cells. The same would also hold true for the cell lines 17–71 and GL-1 where we see lower carbamylation but greater cytotoxicity after treatment with the drugs.

Even though the hepatocyte samples showed greater carbamylation than the cell lines it is not known if this is of clinical relevance with regards to hepatotoxicity caused by lomustine since carbamylation levels have not been measured in dogs that show hepatotoxicity after lomustine treatment. Carbamylation of proteins may interfere with the proper functioning or replication of the cells which may not necessarily result in cell death. It has also been shown that nitrosoureas which form isocyanates are particularly able to inhibit the polymerization of purified brain tubulin in a dose dependent manner [30]. In a study where rats were given lomustine by oral gavage at 50 mg/kg dose, and sacrificed at different time points, the hepatocytes remained normal at early time points although the bile canaliculi became dilated and contained bile thrombi [31]. In another study in rats that were administered lomustine at 20 mg/kg, hepatocytes were not markedly altered with normal nuclei and cell organelles. The main alteration of the parenchymal cells occurred after the 6th day with modification of the cytoskeletal components and reduction in number of microtubules [32]. It is suggested that the isocyanates carbamylate tubulin in hepatocytes leading to mitotic spindle inhibition and G2 cell cycle arrest or may cause disruption of secretory functions such as bile secretion which in turn results in cholestasis and secondary hepatic injury [32,33]. Thus carbamylation of proteins may not necessarily manifest as immediate cell killing.

For the MGMT gene expression studies, HPRT-1 was used as an endogenous control. Before measuring the MGMT expression, we studied the suitability of HPRT-1 as endogenous control in the canine cell lines and liver samples based on the cycle number (cT). HPRT-1 was always measured within 0.5 cT. We understand that the most accurate way of testing HPRT-1 would be by using another endogenous control and that the cT values may be affected by pipetting errors. However, HPRT-1 has a high cT value (above 26), and so these errors would have minimal effect on the cT value.

The canine lymphoma cell line 17–71 showed low MGMT mRNA expression and enzyme activity (about 90 fmols/mg protein). MGMT activity data from humans show that similar level of activity is seen in brain tissue (about 100 fmols/mg protein) which is one of the organs expressing the lowest level of MGMT activity in the body [17]. The GL-1 cells did not show any detectable MGMT mRNA expression or enzyme activity. It has been reported that while MGMT is expressed in all normal human cell types and tissues, 20%–30% of human tumor or more than 50% of virally transformed cell lines are completely deficient in MGMT expression [33,34]. It is not clear yet if the process of immortalization may be leading to loss of MGMT expression [35]. It is known that lack of expression is not due to deletion of the gene [36] and may possibly be an epigenetic event [37,38].

MGMT induction is known to result from transcriptional activation of the MGMT gene in response to DNA damage [39]. In our gene induction study, the 17–71 cells did not show any significant increase in gene expression after 48 h of exposure to lomustine and its metabolites, while the GL-1 cells still did not show any detectable gene expression at all. Time dependent expression of MGMT gene has been measured after exposure to different types of DNA damages including X-ray treatment and methylating agents, and the maximum induction (2–5 fold) was seen at 24 to 72 h [37]. A major limitation of our MGMT gene expression study is the lack of appropriate positive and negative controls. However we believe that this limitation may be offset by the multiple number of technical replicates used and independent experiments performed.

The hepatocytes obtained from the six dogs showed a 2–3 fold greater MGMT mRNA expression as compared to the canine cell line 17–71. The MGMT enzyme activity measured in the hepatocytes is about 250–350 fmol/mg protein as compared to 90 fmol/mg protein in 17–71 cells. This indicates a positive correlation between the MGMT mRNA expression and the enzyme activity in the canine cell lines and the hepatocytes. In Figure 9, it can be noted that the positive correlation between MGMT gene expression and enzyme activity is due mostly to the point corresponding to the 17–71 cells. This data should be considered as preliminary and the correlation can be strengthened by studying the MGMT levels in more dogs/canine cells. Data from humans also suggest that MGMT activity corresponds with the MGMT protein and RNA level [17]. The average MGMT activity in human liver is reported to be around 600 fmol/mg protein, however there is wide inter-individual variation [17,40]. In our MGMT activity assay in hepatocytes, there was over 70% enzymatic cleavage of the substrate oligo (Figure 7). This may indicate a sub-optimal enzyme-substrate ratio which could lead to a lower rate of reaction. It is suggested that enzyme reactions be conducted under initial rate conditions with total consumption of substrate less than 20% [41]. Hence it is possible that the MGMT activity measured in the canine hepatocytes in our study is lower than the actual values.

In our previous study, we have shown that lomustine and its metabolites showed a time and concentration dependent toxicity in canine lymphoma cell lines 17–71 and GL-1 [22]. However the canine hepatocytes did not show any detectable cell killing even 6 days after exposure to the drugs. When the toxic O^6^ guanine adduct was measured in the cells after exposure to lomustine and the metabolites, the canine cell lines showed higher levels of the adduct as compared to the hepatocytes. It is known that MGMT directly repairs the O^6^ adducts in a rapid stoichiometric reaction. In our MGMT study, we saw that the hepatocytes had 2–3 fold greater enzyme activity compared to the 17–71 cell line. Correlating all this data together it is plausible that higher MGMT enzyme activity may be a reason for the lower levels of O^6^ adducts detected in the hepatocyte samples. The higher level of MGMT may cause repair of adducts, and hence contributing to the lower cytotoxicity of lomustine and its metabolites in hepatocytes. However this interpretation must be viewed with caution, as no significant difference in the O^6^ adduct levels between 17–71 cells and GL-1 cells was noted even though we were unable to detect any MGMT activity in the GL-1 cells. Also lomustine and its metabolites form multiple adducts at the O^6^ guanine position out of which we chose the O^6^ hydroxyethyldeoxyguanosine as a marker or indicator of the genotoxic potential of the compounds. It should be noted that MGMT is only one of the several ways in which alkylation damage in DNA is repaired by the cell. Other pathways such as base excision repair and mismatch repair are also known to repair damaged DNA depending on the stage and type of the damage. Thus it may also be possible that the lower O^6^ hydroxyethyldeoxyguanosine measured in the hepatocytes may be due to lower formation or a DNA repair mechanism other than MGMT.

## 6. Conclusions

In this study, we show that lomusine and its metabolites form similar levels of the DNA adducts N^7^ hydroxyethylguanine and O^6^ hydroxyethyldeoxyguanosine which corresponds to the similar cytotoxicity of these compounds. In terms of carbamylation, lomustine showed greater carbamylation in the canine hepatocytes and lymphoma cell lines. Thus if carbamylation of proteins is indeed one of the reasons for the side effects of lomustine, then using the metabolites instead may be a better therapeutic option. Additionally, we also show that MGMT mRNA expression in 17–71 cells and canine hepatocytes correlates with the MGMT enzyme activity in these cells.

## Supplementary Materials

Supplementary File 1

## Abbreviations

BCA: Bicinchoninic acid
bp: Base pair
CBL: Carbamylated lysine
Cis-4: Cis-4-hydroxylomustine
DMEM: Dulbecco’s modified Eagle medium
ELISA: Enzyme-linked immunosorbent assay
FBS: Fetal bovine serum
HEX: Hexachloro-fluorescein
LC/MS/MS: Liquid chromatography-tandem mass spectrometry
MGMT: O^6^methylguanine DNA methyltransferase
MRM: Multiple reaction monitoring
Oligo: Oligonucleaotide
PBMC: Peripheral blood mononuclear cells
PBS: Phosphate buffered saline
RT-PCR: Real time polymerase chain reaction
SD: Standard deviation
Trans-4: Trans-4-hydroxylomustine
