# Supplementary material for: Alkylation and Carbamylation Effects of Lomustine and Its Major Metabolites and MGMT Expression in Canine Cells
Source: Vet Sci. 2015 Apr 24;2(2):52–68. doi: 10.3390/vetsci2020052 (PMC5644621; doi:10.3390/vetsci2020052)
Supplement: Supplementary File 1 [file vetsci-02-00052-s001.docx]

Supplemental Data


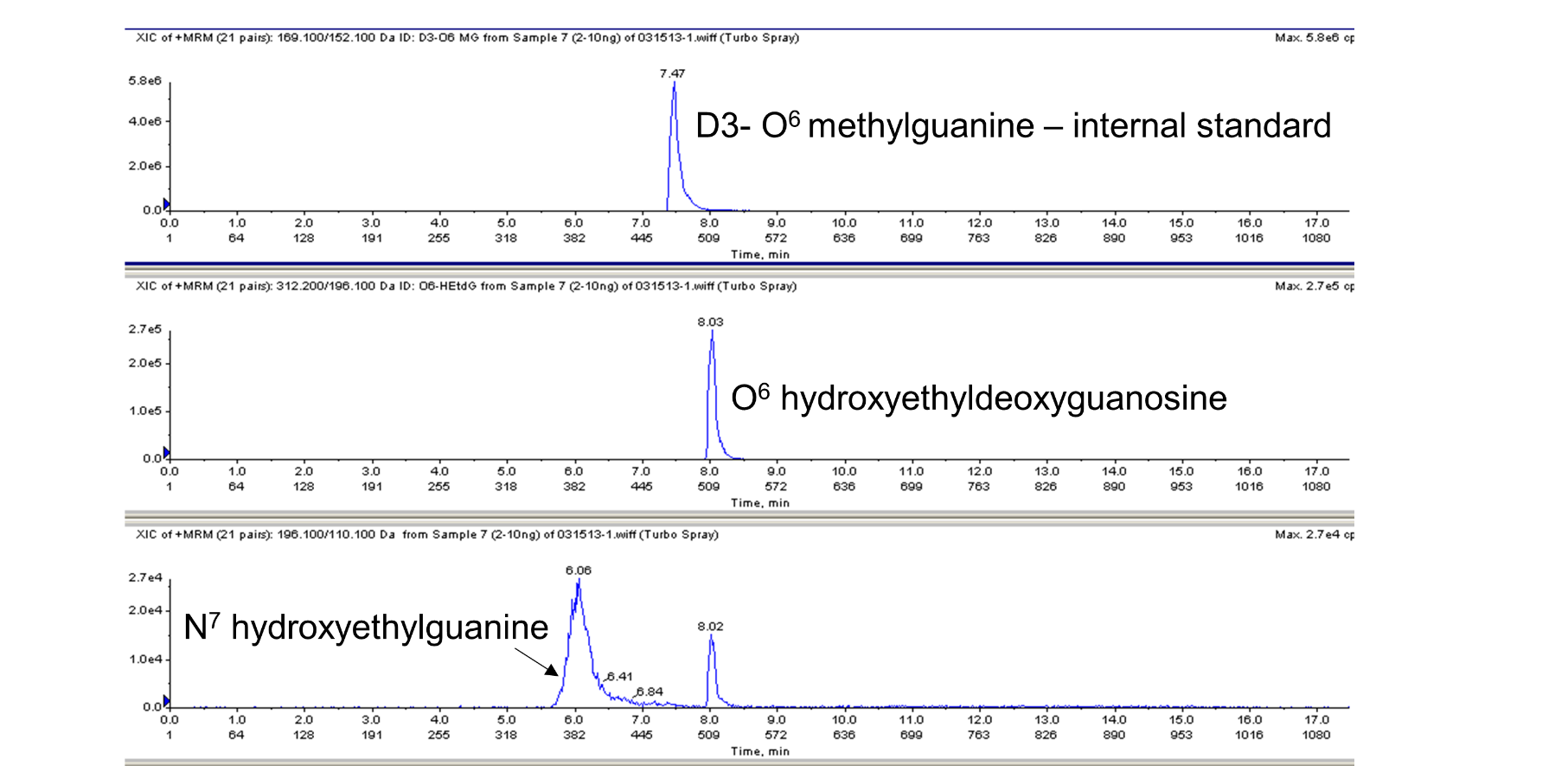


**Figure S1.** LC/MS/MS Multiple Reaction Monitoring (MRM) analysis for O^6^ hydroxyethyldeoxyguanosine, N^7^ hydroxyethylguanine and D3-O^6^ methylguanine.


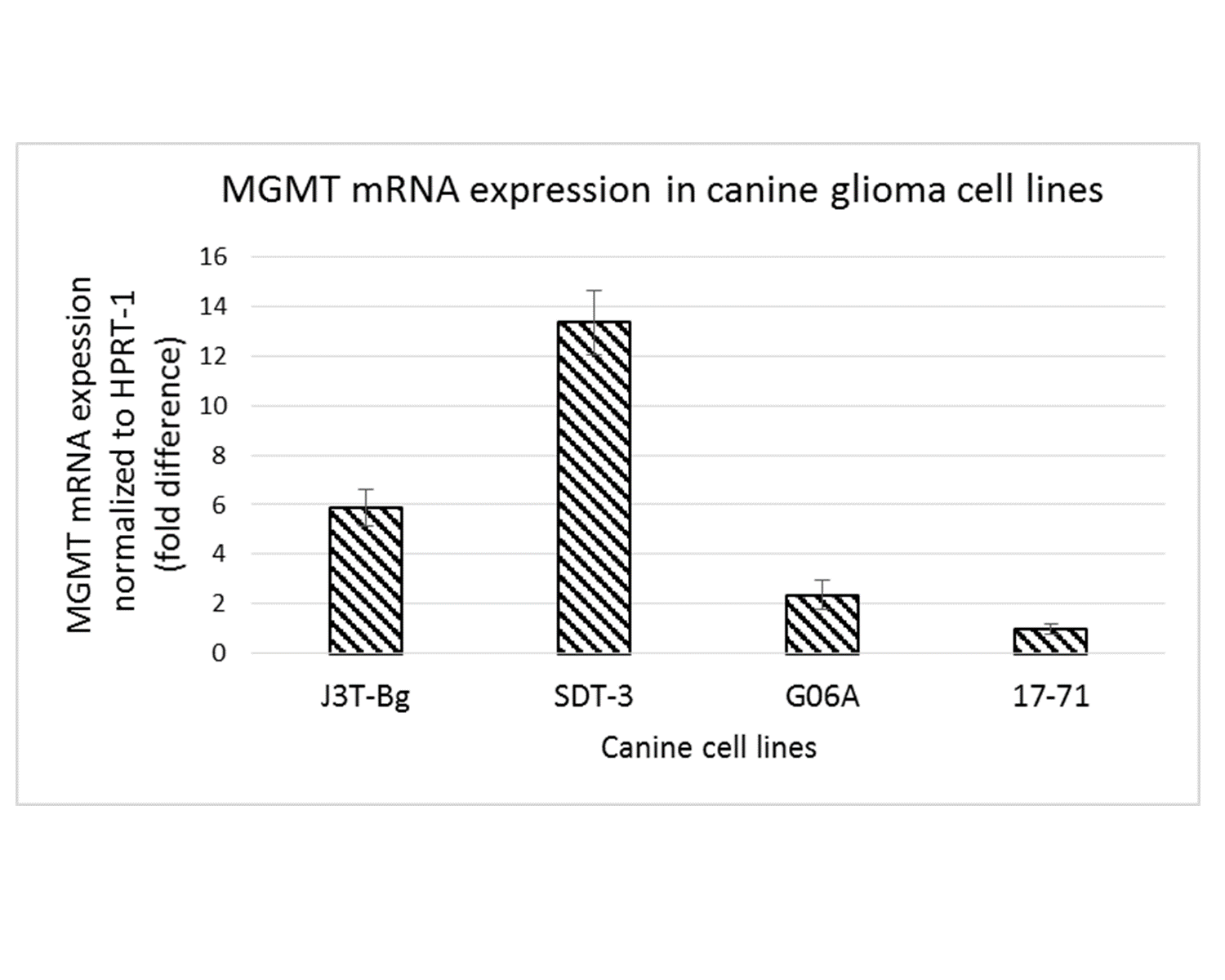


**Figure S2.** Graph showing MGMT mRNA expression in canine glioma cell lines J3T-Bg,
SDT-3 and G06A and canine lymphoma cell line 17–71.


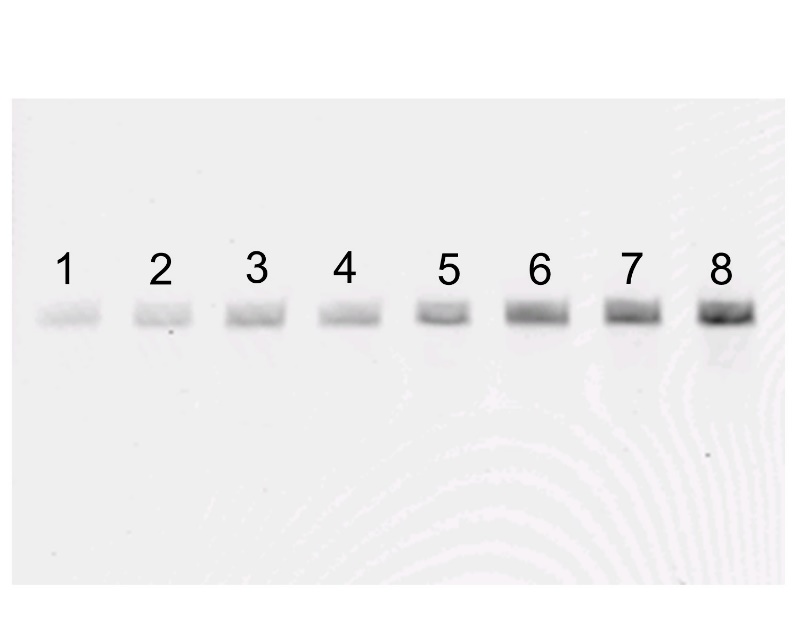


**Figure S3.** Serially diluted oligo from 1–200 fmols to detect LOD and LOQ of the method. Amount of oligo in each lane is as follows: Lane 1 = 1 fmol, lane 2 = 5 fmols, lane 3 = 10 fmols, lane 4 = 20 fmols, lane 5 = 50 fmols, lane 6 = 100 fmols, lane 7 = 150 fmols and lane
8 = 200 fmols.


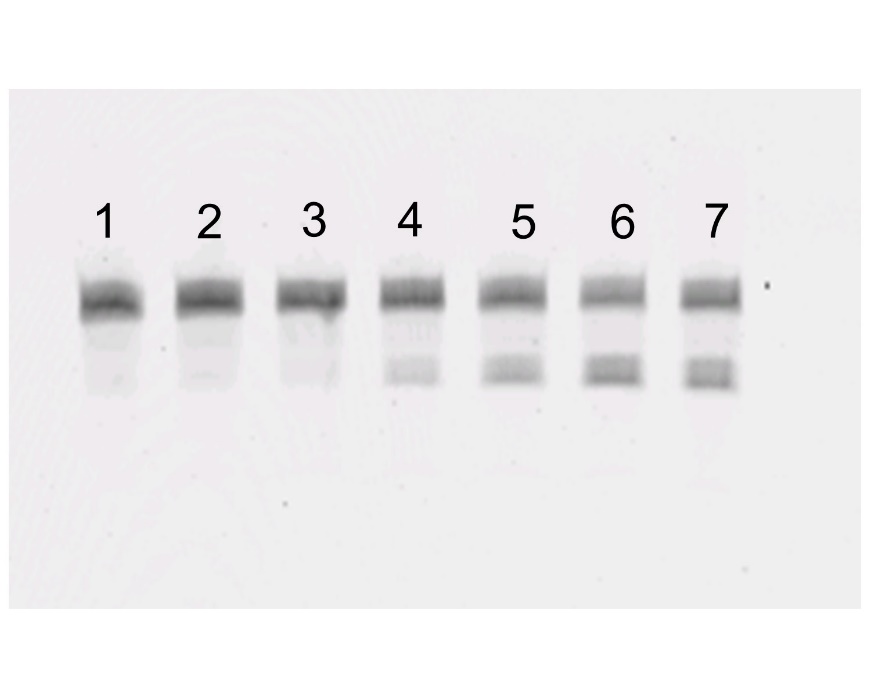


**Figure S4.** Oligo treated with increasing concentration of human recombinant MGMT. Lane 1 represents untreated oligo. Oligos in lane 2–7 were treated with MGMT at 5, 10, 20, 50, 100 and 200 ng/mL concentration. Upper and lower bands represent 18 bp substrate and 10 bp cleavage product, respectively.
